# Knowledge, Attitudes, and Practices of Malaysian Pharmacists Regarding AI Adoption: Cross-Sectional Study

**DOI:** 10.2196/101650

**Published:** 2026-10-20

**Authors:** Chee Tao Chang, Xin Jie Lim, Jason Choong Yin Lee, Chii Chii Chew, Yen Jun Wong, Huan-Keat Chan, Chern Choong Thum, Kar Meng Yew, Shiau Hueh Yeow, Philip Rajan

**Affiliations:** 1 Clinical Research Centre Hospital Raja Permaisuri Bainun Institute for Clinical Research Ipoh, Perak Malaysia; 2 Clinical Research Centre Hospital Raja Permaisuri Bainun Ipoh, Perak Malaysia; 3 Perak Pharmaceutical Services Division Ipoh, Perak Malaysia; 4 School of Pharmacy Monash University Malaysia Subang Jaya, Selangor Malaysia; 5 Centre for Clinical Trials Institute for Clinical Research Setia Alam, Selangor Malaysia; 6 Ministry of Communications Putrajaya Malaysia; 7 Klinik Kesihatan Kampar Ministry of Health Malaysia Kampar, Perak Malaysia; 8 Pharmacy Division BIG CARING Group Klang, Selangor Malaysia

**Keywords:** artificial intelligence, AI, pharmacy, knowledge, attitude, practice, Malaysia

## Abstract

**Background:**

AI is increasingly integrated into health care systems worldwide, with growing implications for pharmacy practice. Although studies have reported general perceptions of AI, gaps between awareness and practical implementation remain evident, particularly in middle-income settings.

**Objective:**

This study aimed to assess the baseline knowledge, attitudes, and practices (KAP) of Malaysian pharmacists regarding AI adoption in pharmacy practice and identify factors associated with these domains.

**Methods:**

A cross-sectional study was conducted using a self-administered online questionnaire distributed to registered pharmacists practicing in Malaysia. The instrument comprised 12 knowledge items, 10 attitude items, and 5 practice items and underwent expert review and pilot testing. KAP scores were categorized using Bloom cutoff points as good (80%-100%), moderate (60%-79%), or poor (<60%). Data were analyzed using R statistical software.

**Results:**

A total of 407 pharmacists completed the survey. The median knowledge score was 75 (IQR 67-92), with 42.3% (172/407) of pharmacists demonstrating good knowledge. The median attitude score was 82 (IQR 76-94), and 64.6% (263/407) of pharmacists exhibited positive attitudes toward AI. However, the median practice score was 56 (IQR 44-68), with 54.3% (221/407) of pharmacists classified as having poor AI use in practice. Statistically significant but weak positive correlations were observed among the 3 domains. Pharmacists with postgraduate qualifications demonstrated significantly higher knowledge scores (*P*=.01).

**Conclusions:**

Malaysian pharmacists demonstrated moderate knowledge and positive attitudes toward AI. However, the practical integration of AI into routine pharmacy workflows remained limited. Strengthening institutional support, structured training, and implementation frameworks may be necessary to translate perceived readiness into clinical adoption.

## Introduction

### Background

AI is increasingly reshaping the pharmaceutical value chain, from accelerating drug discovery and development through advanced data analytics and molecular prediction to enhancing manufacturing efficiency, quality control, and supply chain logistics [1]. In clinical pharmacy, AI supports personalized medicine, clinical decision-making, and pharmacovigilance, with patient safety emerging as a key driver of adoption.

In particular, generative AI (GenAI) and large language models (LLMs) have shown promise in drug-drug interaction detection, clinical decision support, and pharmacovigilance, although performance varies across applications [2]. Collectively, these technologies demonstrate the potential for the early identification of adverse drug events and support safer medication management.

The practical utility of AI in medication safety has been demonstrated through machine learning (ML)–based clinical decision support systems [3]. An evaluation of MedAware, an ML-driven tool, reported a high proportion of clinically valid alerts, many of which were not detectable using traditional rule-based approaches, highlighting the ability of ML to proactively mitigate medication errors, reduce adverse drug events, and generate cost savings [4].

Studies assessing pharmacists’ knowledge, attitudes, and practices (KAP) toward AI have consistently identified gaps between awareness and implementation. Surveys conducted in Saudi Arabia, the United States, and across the Middle East reported generally moderate to good knowledge and positive attitudes [5-7]. However, the practical use of AI tools was limited, largely because of ethical concerns, a lack of formal training, perceived threats to job security, and distrust of AI systems [5-7].

Although AI applications in pharmacy span a broad spectrum, ranging from drug discovery to supply chain management, existing KAP studies have primarily assessed general awareness of AI without adequately evaluating pharmacists’ readiness to adopt AI in medication-related decision-making, such as drug interaction checking, dose adjustment, and pharmacotherapy planning. Hence, understanding pharmacists’ AI literacy and adoption of AI at the point of care is essential.

Despite growing evidence, studies on pharmacists’ engagement with AI in Asian middle-income countries remain scarce. Health care systems in these settings face distinct constraints related to resource availability, digital infrastructure, regulatory environments, and professional training, which may influence AI adoption differently than in high-income contexts.

### Aim

This study aimed to assess the baseline KAP of Malaysian pharmacists toward AI adoption in pharmacy practice, providing a baseline perspective on AI adoption in a middle-income Asian health care system.

## Methods

### Study Design and Study Population

This study used a cross-sectional design using a self-administered online questionnaire, administered to registered pharmacists practicing in Malaysia. The questionnaire was administered in English, and participation was voluntary, with informed consent obtained from all respondents prior to survey completion.

Participants were recruited using nonprobability sampling, combining convenience and snowball sampling approaches. The survey link was disseminated through multiple channels, including professional social media platforms and messaging groups commonly used by pharmacists, websites of relevant pharmacy organizations (where permission was granted), and professional networks to maximize reach. Provisionally and fully registered pharmacists practicing in any sector, including community, hospital, clinical, industry, academia, or regulatory settings, were eligible for inclusion. Pharmacists not practicing in Malaysia, pharmacy students, and retired pharmacists were excluded from the study.

The sample size was estimated using the formula for single-proportion estimation: n=Z^2^P(1−P)/*d*^2^, where Z=1.96 (95% confidence level), *P*=0.50 (a conservative estimate of the expected proportion, adopted in the absence of prior local data to yield the maximum required sample size), and Cohen *d*=0.05 (a margin of error of 5%). The minimum required sample size was 385 participants. To account for potential nonresponse and incomplete questionnaires, the target sample size was increased to 400 pharmacists.

### Study Instrument

The questionnaire was developed in English to reflect its common professional and academic use among Malaysian pharmacists and to ensure consistency with the predominantly English-language AI literature. Item development was guided by existing studies assessing pharmacists’ KAP toward AI, with adaptations from previously published instruments [5-7]. To ensure contemporary relevance, additional insights were drawn from scholarly reviews on GenAI and LLMs in pharmacy and clinical practice [1,2,4,8].

The conceptual framework for the questionnaire was grounded in established theoretical models. The knowledge domain was structured according to the Bloom taxonomy of cognitive complexity, assessing AI fundamentals, applications, and critical analysis, consistent with the framework proposed previously [9]. The attitude domain was informed by the extended technology acceptance model, which originated from the technology acceptance model [10], and subsequent developments that encompassed perceived usefulness, performance expectancy, effort expectancy, and future outlook [11]. The practice domain assessed actual behavioral engagement with AI tools, consistent with the behavioral outcome component of the theory of planned behavior [12], complementing the attitude domain, which captured the motivational antecedents of behavior.

Within the knowledge domain, items were organized according to 3 cognitive levels of the Bloom taxonomy: foundational understanding of AI concepts and terminology (K1-K2); application of AI in pharmacy contexts, including drug interaction detection, medication error reduction, and adherence monitoring (K5-K9); and evaluation of AI limitations and risks, including training data bias, hallucinations, and knowledge cutoff dates (K10-K12). Items K3 and K4 assessed the conceptual distinction between predictive and GenAI. The attitude domain was mapped to key constructs of the extended technology acceptance model, encompassing perceived usefulness (A1-A4); performance expectancy (A5-A6); effort expectancy (A7-A8); and future outlook, including willingness to adopt AI and support for its integration into pharmacy education (A9-A10). The practice domain assessed the frequency of AI use in direct patient care activities, including drug information retrieval, drug interaction checking, patient counseling, and pharmacotherapy support (P1-P4), consistent with the theory of planned behavior. Item P5 captured pharmacists’ experience of encountering AI-generated errors in practice.

Content validity was established through a 3-round expert review process, in which an initial pool of items adapted from existing instruments [5-7,13] was refined into a preliminary questionnaire comprising 15 knowledge items, 13 attitude items, and 5 practice items. The panel comprised 6 experts purposively selected based on their professional expertise relevant to the study topic, spanning clinical pharmacy research, pharmacy policy and administration, pharmacy academia, clinical medicine, and digital health policy. Specifically, the panel consisted of 2 senior research pharmacists, 1 senior pharmacist in policy and administration, 1 university pharmacy lecturer, 1 research medical officer, and 1 medical physician holding a policy role at the Ministry of Communications. This multidisciplinary composition ensured that the instrument was evaluated from multiple perspectives pertinent to AI adoption in pharmacy practice.

Following expert review, pretesting, and a pilot study involving 30 pharmacists, 3 items were removed from each of the knowledge and attitude domains due to redundancy or suboptimal performance, resulting in a final instrument with 12 knowledge items, 10 attitude items, and 5 practice items. Reliability testing after refinement demonstrated variable internal consistency across domains, with Cronbach α values of 0.63 for the knowledge domain, 0.93 for the attitude domain, and 0.81 for the practice domain. Although the attitude and practice domains demonstrated good to excellent internal consistency, the knowledge domain had an α value below the conventional threshold of 0.70 [14].

Knowledge items were presented in a true or false format and scored dichotomously as correct or incorrect. Attitude items were rated on a 5-point Likert scale ranging from strongly disagree to strongly agree, and practice items were rated on a 5-point frequency scale ranging from never to always. Domain scores were computed as the sum of item scores, converted to a percentage of the maximum possible score, and subsequently categorized using Bloom cutoff points.

### Data Collection

Data were collected using a self-administered online questionnaire developed with Google Forms (Alphabet Inc). A unique survey link was disseminated through multiple electronic channels, including direct email invitations where feasible, mobile messaging apps such as WhatsApp (Meta Platforms Inc) groups relevant to pharmacists, and professional social media platforms. Permission was obtained from group or page administrators prior to distribution. The questionnaire required approximately 5 to 10 minutes to complete. Data collection was conducted from October 21, 2025, to February 3, 2026.

### Statistical Analysis

Data analyses were performed using R (version 4.4.1; R Foundation for Statistical Computing). Statistical significance was defined as a 2-sided *P* value of <.05.

Descriptive statistics were generated to summarize the sociodemographic characteristics and KAP scores of the study population. Categorical variables were reported as frequencies (percentages), while continuous variables were presented as medians (IQRs) due to the nonnormal distribution of the data. The KAP scores were categorized using Bloom cutoff points, with knowledge classified as good (80%-100%), moderate (60%-79%), or poor (<60%); attitudes as positive (80%-100%), neutral (60%-79%), or negative (<60%); and practice as good (80%-100%), fair (60%-79%), or poor (<60%) [15]. The distribution of responses for individual items was visualized using stacked bar charts.

To identify factors associated with KAP scores, univariable and multivariable linear regression models were constructed. Univariable regression was first used to estimate the unadjusted association between sociodemographic factors (age, sex, sector, working experience, and education level) and the KAP domain scores.

Prior to fitting the multivariable models, multicollinearity among candidate predictors was assessed using variance inflation factors (VIFs). Initial diagnostics revealed severe multicollinearity between age (VIF=16.13) and work experience (VIF=16.39), exceeding the conventional threshold of VIF>5. To resolve this and ensure model stability, age was removed from the multivariable models, as work experience was deemed conceptually more proximal to AI-related knowledge, attitude, and practice outcomes than chronological age. Following its removal, multicollinearity was resolved, with all remaining predictors demonstrating excellent stability (all final VIFs <1.11).

The final multivariable models included sex, sector (government vs private), work experience (in years), and education level as covariates. Regression results were visualized using forest plots displaying β coefficients and 95% CIs across the 3 domains.

Model assumptions were formally evaluated. The assumption of homoscedasticity was satisfied across all models, with Studentized Breusch-Pagan tests yielding nonsignificant results for knowledge (*P*=.09), attitude (*P*=.31), and practice (*P*=.91), indicating stable and uniformly distributed residual variance. Shapiro-Wilk testing indicated that residuals of the practice model approximated normality (*P*=.10), whereas the residuals of the knowledge and attitude models showed statistically significant deviations from normality (both *P*<.001). Given this departure from normality, heteroscedasticity-consistent robust SEs (heteroscedasticity-consistent estimator 3 [HC3]) were applied across all models to ensure valid inference independent of the residual distribution.

The interrelationships between the KAP domains were evaluated using Spearman rank correlation coefficients. The strength of correlations was interpreted as negligible (0.00-0.19), weak (0.20-0.39), moderate (0.40-0.59), strong (0.60-0.79), or very strong (0.80-1.00) [16]. Spearman rank correlation was used given the nonnormal distribution of KAP domain scores. As all 3 domains were operationalized as composite percentage scores prior to analysis and Spearman correlation operates on ranked values rather than raw scores, this approach is inherently robust to differences in underlying item formats and distributional assumptions, making it appropriate for assessing associations across these heterogeneous domains.

### Ethical Considerations

This study was registered with the National Medical Research Registry (NMRR; ID-25-02129-WPP [IIR (investigator-initiated research)]), and ethics approval was obtained from the Medical Research and Ethics Committee (MREC; 25-02129-WPP[2]). Before accessing the questionnaire, potential participants were directed to an online participant information sheet detailing the study objectives, methodology, estimated completion time, potential risks and benefits, the voluntary nature of participation, and measures to ensure confidentiality and anonymity. An electronic version of the consent form was signed online, and informed consent was obtained from all participants. No personally identifiable information, including names or national identification numbers, was collected. Participants’ personal phone numbers and email addresses were also not collected at any stage, and no contact details were obtained from administrative departments for survey dissemination. Participation was voluntary, and nonparticipation carried no consequences. Participants did not receive any compensation for their participation in this study.

## Results

A total of 410 pharmacists consented to participate in the study, and 407 (99.3%) completed the survey and were included in the analysis. The median age of respondents was 36 (IQR 31-40) years, with the majority being female (301/407, 74%). Most participants were employed in the public sector (309/407, 76%), predominantly in public hospitals (211/407, 51.8%) and primary health clinics (76/407, 18.7%). The majority held a bachelor’s degree (336/407, 82.6%), while 15.5% (63/407) had attained a master’s degree (Table 1).

**Table 1:** Characteristics of study participants (N=407).

Characteristics			Participants
Age (years), median (IQR)			36 (31-40)
**Sex, n (%)**			
	Female	301 (74)	
	Male	106 (26)	
**Employment sector, n (%)**			
	Public sector (government)	309 (75.9)	
	Private sector	88 (21.6)	
	Self-employed (own business)	8 (2)	
	Nongovernmental organizations	2 (0.5)	
Work experience (years), median (IQR)			12 (7-16)
**Highest education level, n (%)**			
	Bachelor’s degree	336 (82.6)	
	Master’s degree	63 (15.5)	
	PhD	8 (1.9)	
**Workplace setting, n (%)**			
	Hospital (public)	211 (51.8)	
	Community pharmacy (private)	78 (19.2)	
	Health clinic (public)	76 (18.7)	
	Pharmaceutical service division (public)	19 (4.7)	
	Pharmaceutical industry (private)	9 (2.2)	
	Other settings (eg, private clinic and private hospital)	9 (2.2)	
	University, teaching hospital, or research institution	5 (1.2)	

The median knowledge score was 75 (IQR 67-92), with 42.3% (172/407) of respondents classified as having good knowledge of AI. The median attitude score was 82 (IQR 76-94), and 64.6% (263/407) of participants demonstrated a positive attitude. In contrast, practice scores were comparatively lower, with a median score of 56 (IQR 44-68). Of the 407 respondents, 221 (54.3%) were categorized as having poor AI use in pharmacy practice.

Item-level analysis of the knowledge domain indicated that 53.3% (217/407) of respondents incorrectly identified the distinction between predictive AI systems and GenAI chatbots (K3), and 48.4% (n=197) failed to recognize the concept of AI hallucinations (K11). In the practice domain, 50.9% (207/407) reported rarely or never encountering incorrect information generated by AI tools in health care practice (P5; Figure 1).

**Figure 1:**
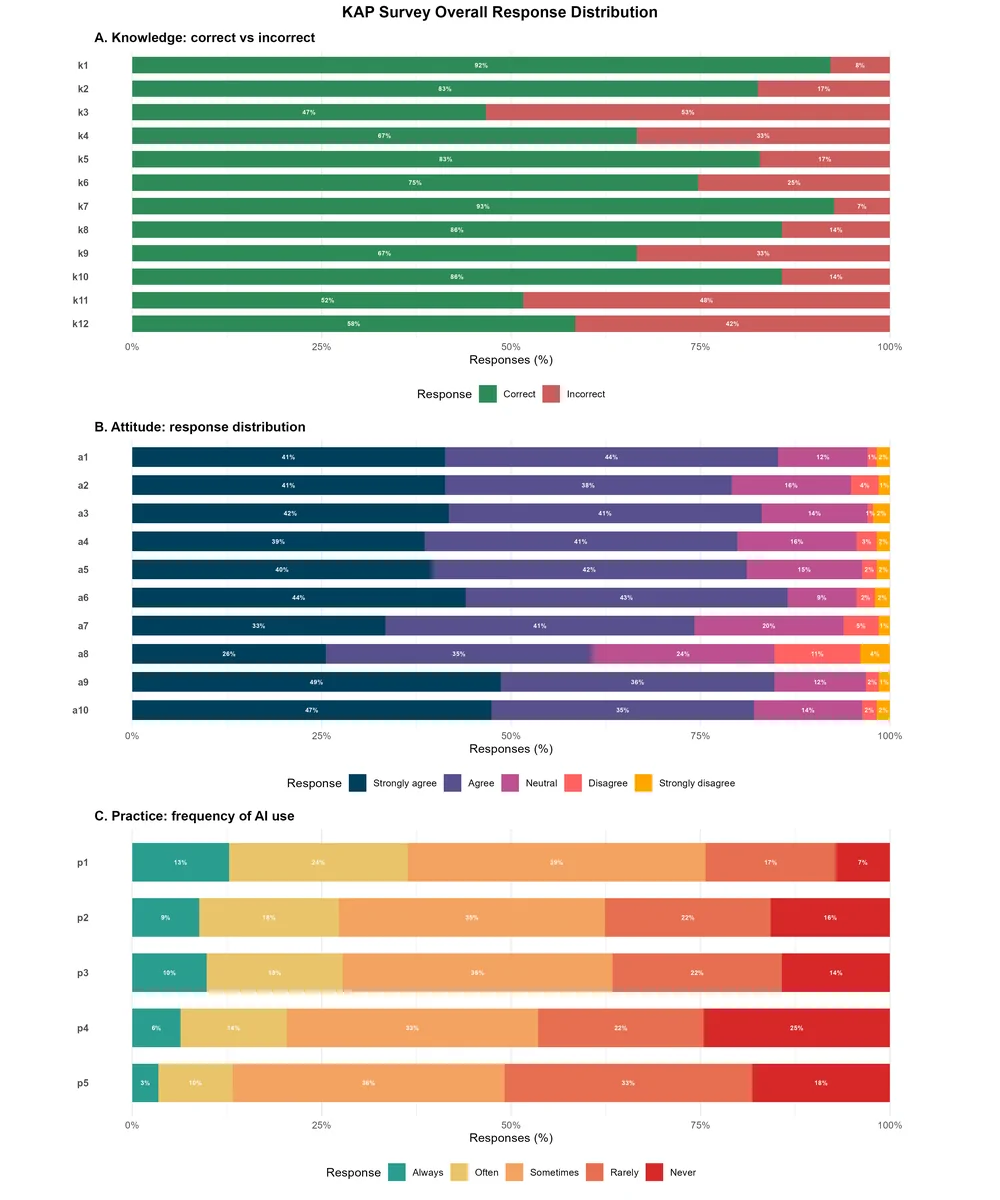
Item-level responses across knowledge, attitude, and practice domains (KAP).

Correlation analyses demonstrated statistically significant positive associations among knowledge, attitude, and practice scores. Knowledge scores were weakly correlated with attitude scores (*r*=0.36; *P*<.001), as were attitude scores with practice scores (*r*=0.28; *P*<.001). A weak positive correlation was also observed between knowledge and practice scores (*r*=0.25; *P*<.001; Table 2).

**Table 2:** Correlation between knowledge, attitude, and practice scores.

Measures	Correlation coefficient (*r*_s_)	*P* value
Knowledge and attitude	0.36	<.001
Knowledge and practice	0.25	<.001
Attitude and practice	0.28	<.001

Multivariable linear regression analysis revealed that pharmacists with a master’s degree demonstrated significantly higher AI knowledge scores than those holding a bachelor’s degree (*P*=.01). No statistically significant associations were observed between the examined variables and either attitude or practice scores (Figure 2; Multimedia Appendices 1-3).

**Figure 2:**
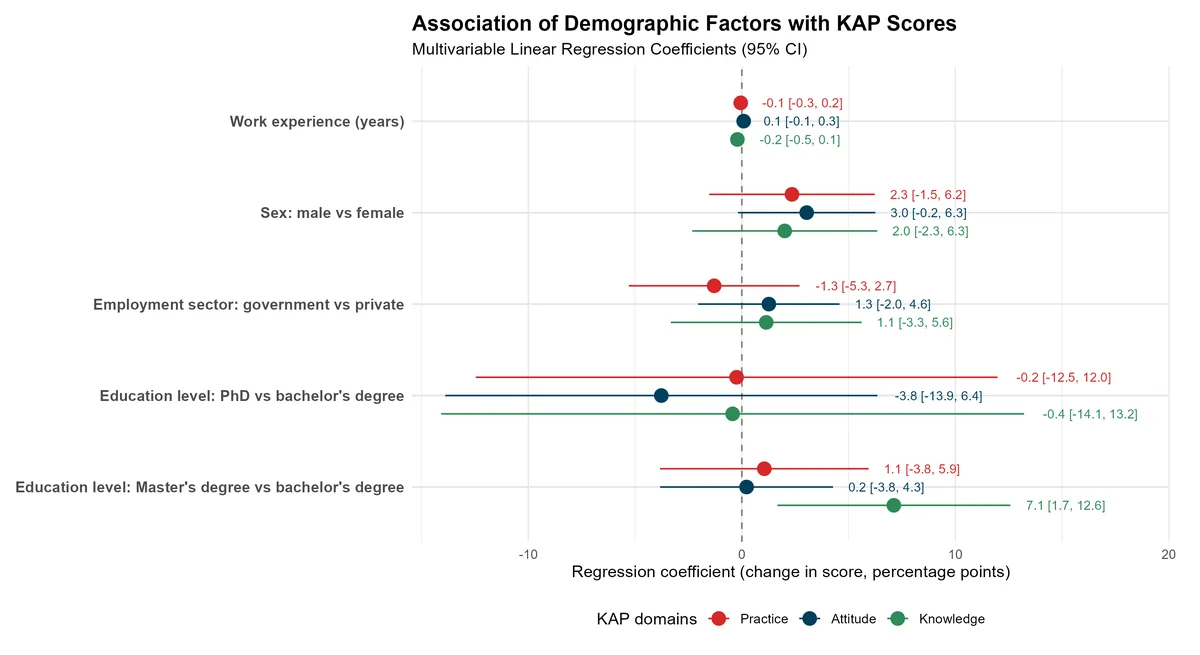
Multivariable linear regression analysis of factors associated with knowledge, attitudes, and practices (KAP) domains.

## Discussion

### Principal Findings

Overall, pharmacists demonstrated a moderate level of knowledge and positive attitudes toward AI, indicating both intellectual engagement with the technology and openness to its potential role in health care delivery. However, this observed readiness was not reflected in routine clinical application, as engagement with AI-related tools in pharmacy practice remained limited. The observed disparity suggests a gap between pharmacists’ receptiveness to AI use in pharmacy practice and its actual adoption, highlighting the complexity of translating positive perceptions into clinical practice.

These findings are broadly consistent with, yet contextually distinct from, international studies. In the United States, Gustafson et al [5] found that although 82.5% of pharmacists were familiar with AI, only 38.7% had used AI in practice and 34.9% expressed distrust, indicating that the gap between awareness and adoption persists even in digitally advanced settings. Likewise, Jaber et al [6] reported similar levels of AI knowledge (44.8%) but a lower proportion of positive attitudes (49.1%) among pharmacists across the Middle East than observed in the present study. Similarly, Alghitran et al [7] found that although 64.1% of Saudi pharmacists held positive attitudes toward ChatGPT, only 24.3% used AI regularly and 92% lacked formal training. Collectively, these findings suggest that the gap between AI readiness and actual clinical adoption is not unique to Malaysia but is consistently observed across health care systems, particularly where formal AI training, institutional support, and implementation strategies remain underdeveloped.

Although more than three-quarters of respondents demonstrated good or moderate overall knowledge, item-level analysis revealed specific deficiencies in understanding the distinction between predictive and GenAI systems and in recognizing the phenomenon of AI hallucinations. This pattern reflects surface-level familiarity, whereby pharmacists recognize AI tools but lack deeper insight into their underlying mechanisms and associated risks. Similar findings have been reported internationally, where studies have consistently reported high levels of AI awareness alongside moderate technical understanding and limited operational depth [7,13,17-19]. Consequently, AI literacy among Malaysian pharmacists appears largely conceptual, which may limit their capacity to critically appraise and safely apply AI tools in professional practice.

The inability of more than half of respondents to distinguish predictive AI from GenAI indicates a fundamental conceptual gap that may lead to inappropriate tool selection, while limited awareness of AI hallucinations raises concerns about automation bias and patient safety, particularly in high-risk tasks such as drug interaction screening, dose calculation, and pharmacovigilance. The finding that most respondents rarely encountered incorrect AI-generated information likely reflects limited AI exposure rather than AI reliability, as those with minimal experience may be less able to recognize erroneous outputs. Together, these findings underscore the need for continuing professional development programs that strengthen AI literacy and emphasize the critical appraisal of AI outputs, recognition of GenAI limitations, and use of appropriate verification practices in clinical workflows.

Educational attainment emerged as the primary factor associated with stronger AI knowledge, with pharmacists holding postgraduate qualifications demonstrating greater proficiency. This association (*P*=.01) likely reflects increased exposure to research-intensive environments where advanced digital tools are more commonly used [7,18]. In contrast to findings from high-income settings where younger pharmacists were more likely to report greater AI engagement than older age groups [5], no significant age-related association was observed across any KAP domain in the present study, based on univariable regression analysis. This suggests that, within the Malaysian context, AI proficiency may be shaped more by professional exposure and access to training opportunities than by generational differences in technology adoption [20].

The highly positive attitudes toward AI observed in this study reflect a high level of psychological readiness among Malaysian pharmacists to engage with AI technologies, despite gaps in technical knowledge. Similar attitudes have been reported across health care professions, with clinicians expressing confidence in AI’s supportive role in practice while simultaneously acknowledging concerns related to trust, ethics, and preparedness [5,6,21]. This perspective aligns with a broader emerging consensus that AI is increasingly regarded as a complementary component of future clinical decision-making rather than a disruptive replacement, even in settings where formal AI training remains limited [22].

Importantly, attitudes toward AI in the present study were not shaped by demographic or professional characteristics, indicating that receptiveness to AI is broadly shared across the Malaysian pharmacy workforce. This contrasts with findings from other settings, where attitudes and knowledge related to AI vary according to background, training, or sociodemographic factors [13,21,23]. The absence of such stratification in the present study suggests a more uniform professional consensus regarding the role of AI within Malaysian pharmacy practice. This consistency reflects a shared recognition that AI integration is becoming an essential component of contemporary health care practice, underscoring the need for pharmacy practice to evolve alongside the ongoing digitalization of medicine [24].

However, more than half of the respondents demonstrated poor levels of AI use in pharmacy practice. Similar gaps have been reported elsewhere, where high awareness has not translated into sustained professional use due to the absence of formal training, institutional guidance, and approved operational frameworks [5,7]. Studies have similarly reported limited AI use despite generally positive attitudes, often attributed to limited access, lack of organizational readiness, and uncertainty regarding governance and accountability [25,26]. The weak correlations observed between knowledge, attitudes, and practice further suggest that individual readiness alone is insufficient to drive AI adoption, suggesting that structural and contextual factors may also influence AI adoption beyond individual readiness, although they were not directly measured in this study.

Malaysia’s National Guidelines on Artificial Intelligence Governance and Ethics (AIGE) provide only voluntary, cross-sector guidance, while health care–specific AI regulations, accountability frameworks, and institutional support remain lacking, potentially discouraging pharmacists from adopting AI in clinical practice. Additionally, the predominance of public sector pharmacists, together with variable digital infrastructure across health care settings, may further limit access to and integration of AI tools. As AI literacy is also not yet formally embedded within Malaysian pharmacy curricula, closing the knowledge-practice gap will likely require coordinated regulatory, organizational, infrastructural, and educational interventions.

A majority of respondents reported rarely encountering incorrect information generated by AI tools, suggesting a possible underestimation of their limitations. LLMs can produce inaccurate or biased clinical outputs, particularly without safeguards or domain-specific tuning, and the lack of users’ experience may conceal these risks [2]. Similar patterns have been observed in other health care settings, where professionals demonstrate optimism toward AI but limited awareness of its potential errors and risks in practice [27,28]. Although AI can identify errors missed by traditional systems, it also introduces validity challenges that require active professional oversight [4]. Without sufficient technical literacy and verification skills, pharmacists may be susceptible to automation bias, highlighting the importance of embedding critical appraisal and verification processes into routine pharmacy workflows [29,30].

This study has several limitations that should be acknowledged. First, its cross-sectional design captured participants’ KAP at a single time point, precluding causal inference and limiting the ability to assess changes in pharmacists’ KAP toward AI over time. Second, the use of nonprobability sampling via digital channels, including WhatsApp groups and social media platforms, may introduce selection and response bias, as pharmacists who are already digitally engaged are more likely to encounter and complete an online survey. Although this recruitment strategy was necessary to enable wider reach, the findings may not be fully representative of pharmacists practicing in rural or less digitally integrated settings and should therefore be interpreted as a baseline estimate. Third, AI-related practices were self-reported, which may not reflect actual use in routine pharmacy workflows, and the absence of objective validation, such as workflow observation or institutional audit data, limits the reliability of practice assessments. Fourth, the Cronbach α for the knowledge domain was below the conventional threshold of 0.70, which likely reflects the intentional heterogeneity of items spanning multiple cognitive levels rather than inadequate instrument quality. Nevertheless, this warrants cautious interpretation of the findings for the knowledge domain. Finally, the classification of KAP scores using Bloom cutoff points represents an arbitrary threshold that has not been formally validated for AI literacy assessment in pharmacists and carries inherent risks of misclassification and reduced statistical power. However, all inferential analyses were conducted on continuous domain scores, with Bloom categorization applied descriptively only to facilitate interpretation and cross-study comparability.

Future research could use qualitative designs to better understand the contextual factors influencing real-world AI adoption, particularly the barriers and facilitators shaping implementation in routine pharmacy practice. Subsequently, intervention-based studies evaluating structured implementation pilots would be valuable in determining effective strategies for translating positive attitudes and knowledge into meaningful integration into practice.

### Conclusions

Malaysian pharmacists demonstrated moderate knowledge and positive attitudes toward AI in pharmacy, while practical adoption remained limited. These findings indicate that targeted strategies are needed to bridge the gap between AI readiness and sustained real-world use. These strategies may include structured training programs for capacity building, organizational support, and clear regulatory and ethical guidelines. User-friendly digital infrastructure and a supportive practice environment are also essential enablers of effective AI adoption in pharmacy practice, contributing to improved pharmacy workflow efficiency and, importantly, enhanced patient-centered care delivery.

## Data Availability

The datasets generated during and/or analyzed in the current study are available from the corresponding author upon reasonable request.

## Appendix

Multimedia Appendix 1 Univariable and multivariable linear regression analyses of factors associated with knowledge scores among Malaysian pharmacists.

Multimedia Appendix 2 Univariable and multivariable linear regression analyses of factors associated with attitude scores among Malaysian pharmacists.

Multimedia Appendix 3 Univariable and multivariable linear regression analyses of factors associated with practice scores among Malaysian pharmacists.

## Abbreviations

AIGE: Artificial Intelligence Governance and Ethics
GenAI: generative AI
HC3: heteroscedasticity-consistent estimator 3
KAP: knowledge, attitudes, and practices
LLM: large language model
ML: machine learning
MREC: Medical Research and Ethics Committee
NMRR: National Medical Research Registry
VIF: variance inflation factor
