# Supplementary material for: Knowledge, Attitudes, and Practices of Malaysian Pharmacists Regarding AI Adoption: Cross-Sectional Study
Source: JMIR Form Res. 2026 Oct 20;10:e101650. doi: 10.2196/101650 (PMC13732175; doi:10.2196/101650)
Supplement: Supplementary file 1 [file formative_v10i1e101650_app1.docx]

Table S1. Univariable and Multivariable Linear Regression Analysis of Factors Associated with Knowledge Scores Among Malaysian Pharmacists

|  | **Simple (Unadjusted)** | | | | **Multiple (Adjusted)** | | |
| --- | --- | --- | --- | --- | --- | --- | --- |
| **Characteristic** | **N** | **Beta** | **95% CI** | **p-values** | **Beta** | **95% CI** | **p-values** |
| **Age (Years)** | 407 | -0.13 | -0.39, 0.14 | 0.342 |  |  |  |
| **Gender** | 407 |  |  | 0.368 |  |  | 0.363 |
| *Female* |  | — | — |  | — | — |  |
| *Male* |  | 2.0 | -2.3, 6.3 |  | 2.0 | -2.3, 6.3 | 0.363 |
| **Sector (vs Private)** | 407 |  |  | 0.672 |  |  | 0.616 |
| *Private* |  | — | — |  | — | — |  |
| *Government* |  | 0.95 | -3.5, 5.4 |  | 1.1 | -3.3, 5.6 | 0.616 |
| **Working Experience (Years)** | 407 | -0.10 | -0.38, 0.18 | 0.488 | -0.21 | -0.50, 0.08 | 0.157 |
| **Education Level (vs Bachelor's degree)** | 407 |  |  | 0.074 |  |  | **0.037** |
| *Bachelor's degree* |  | — | — |  | — | — |  |
| *Master's degree* |  | 6.0 | 0.81, 11 |  | 7.1 | 1.7, 13 | **0.011** |
| *PhD* |  | -1.1 | -15, 13 |  | -0.43 | -14, 13 | 0.950 |
| Abbreviation: CI = Confidence Interval | | | | | | | |
