# Supplementary material for: Knowledge, Attitudes, and Practices of Malaysian Pharmacists Regarding AI Adoption: Cross-Sectional Study
Source: JMIR Form Res. 2026 Oct 20;10:e101650. doi: 10.2196/101650 (PMC13732175; doi:10.2196/101650)
Supplement: Supplementary file 2 [file formative_v10i1e101650_app2.docx]

Table S2. Univariable and Multivariable Linear Regression Analysis of Factors Associated with Attitude Scores Among Malaysian Pharmacists

|  | **Simple (Unadjusted)** | | | | **Multiple (Adjusted)** | | |
| --- | --- | --- | --- | --- | --- | --- | --- |
| **Characteristic** | **N** | **Beta** | **95% CI** | **p-values** | **Beta** | **95% CI** | **p-values** |
| **Age (Years)** | 407 | 0.11 | -0.08, 0.30 | 0.267 |  |  |  |
| **Gender** | 407 |  |  | 0.079 |  |  | 0.064 |
| *Female* |  | — | — |  | — | — |  |
| *Male* |  | 2.8 | -0.33, 6.0 |  | 3.0 | -0.18, 6.3 | 0.064 |
| **Sector** | 407 |  |  | 0.548 |  |  | 0.453 |
| *Private* |  | — | — |  | — | — |  |
| *Government* |  | 1.0 | -2.3, 4.3 |  | 1.3 | -2.0, 4.6 | 0.453 |
| **Working Experience (Years)** | 407 | 0.09 | -0.12, 0.29 | 0.403 | 0.09 | -0.13, 0.30 | 0.432 |
| **Education Level** | 407 |  |  | 0.671 |  |  | 0.756 |
| *Bachelor's degree* |  | — | — |  | — | — |  |
| *Master's degree* |  | 0.78 | -3.1, 4.7 |  | 0.22 | -3.8, 4.3 | 0.914 |
| *PhD* |  | -4.0 | -14, 6.1 |  | -3.8 | -14, 6.4 | 0.464 |
| Abbreviation: CI = Confidence Interval | | | | | | | |
