# Supplementary material for: Knowledge, Attitudes, and Practices of Malaysian Pharmacists Regarding AI Adoption: Cross-Sectional Study
Source: JMIR Form Res. 2026 Oct 20;10:e101650. doi: 10.2196/101650 (PMC13732175; doi:10.2196/101650)
Supplement: Supplementary file 3 [file formative_v10i1e101650_app3.docx]

Table S3. Univariable and Multivariable Linear Regression Analysis of Factors Associated with Practice Scores Among Malaysian Pharmacists

|  | **Simple (Unadjusted)** | | | | **Multiple (Adjusted)** | | |
| --- | --- | --- | --- | --- | --- | --- | --- |
| **Characteristic** | **N** | **Beta** | **95% CI** | **p-values** | **Beta** | **95% CI** | **p-values** |
| **Age (Years)** | 407 | -0.02 | -0.25, 0.21 | 0.868 |  |  |  |
| **Gender** | 407 |  |  | 0.192 |  |  | 0.235 |
| *Female* |  | — | — |  | — | — |  |
| *Male* |  | 2.5 | -1.3, 6.4 |  | 2.3 | -1.5, 6.2 | 0.235 |
| **Sector** | 407 |  |  | 0.418 |  |  | 0.524 |
| *Private* |  | — | — |  | — | — |  |
| *Government* |  | -1.6 | -5.6, 2.3 |  | -1.3 | -5.3, 2.7 | 0.524 |
| **Working Experience (Years)** | 407 | -0.04 | -0.29, 0.20 | 0.735 | -0.05 | -0.31, 0.21 | 0.690 |
| **Education Level** | 407 |  |  | 0.952 |  |  | 0.912 |
| *Bachelor's degree* |  | — | — |  | — | — |  |
| *Master's degree* |  | 0.74 | -3.9, 5.4 |  | 1.1 | -3.8, 5.9 | 0.672 |
| *PhD* |  | -0.01 | -12, 12 |  | -0.25 | -12, 12 | 0.969 |
| Abbreviation: CI = Confidence Interval | | | | | | | |
